# Pesticide residues in some herbs growing in agricultural areas in Poland

**DOI:** 10.1007/s10661-015-4997-1

**Published:** 2015-11-26

**Authors:** Elżbieta Malinowska, Kazimierz Jankowski

**Affiliations:** Department of Grassland and Landscape Architecture, Siedlce University of Natural Sciences and Humanities, B. Prusa 14 Street, 08-110 Siedlce, Poland

**Keywords:** Selected herbs, The QuECHERS method, Plant protection product residues, Poland

## Abstract

The aim of this paper was to assess residue content of plant protection products in selected herbs*: Achillea millefolium* L., *Cichorium intybus* L., *Equisetum arvense* L., *Polygonum persicaria* L., *Plantago lanceolata* L., and *Plantago major* L. The study comprises herbs growing in their natural habitat, 1 and 10 m away from crop fields. The herbs, 30 plants of each species, were sampled during the flowering stage between 1 and 20 July 2014. Pesticide residue content was measured with the QuECHERS method in the dry matter of leaves, stalks, and inflorescence, all mixed together. Out of six herb species growing close to wheat and maize fields, pesticide residues were found in three species: *A. millefolium* L., *E. arvense* L., and *P. lanceolata* L. Most plants containing the residues grew 1 m away from the wheat field. Two active substances of fungicides were found: diphenylamine and tebuconazole, and one active substance of insecticides: chlorpyrifos-ethyl. Those substances are illegal to use on herbal plants. Samples of *E. arvense* L. and *P. lanceolata* L. contained two active substances each, which constituted 10 % of all samples, while *A. millefolium* L. contained one substance, which is 6.6 % of all samples.

## Introduction

The use of plant protection products in agriculture results in economic benefits but can be hazardous to the environment, in particular to people and animals (Diez et al. 2006; Łozowicka 2009). Pesticides belong to substances which are the most toxic and are persistent; they do not break down easily, have ability to bio-accumulate, and can be mobile in the environment. They can also become mutagenic, carcinogenic, teratogenic, and allergenic. Pesticides can enter an organism through the digestive system, and even small amounts can be harmful if their intake lasts longer (Kroes et al. 2000; Gorrido et al. 2003). Human food and livestock feed should not contain pesticide residues over the maximum residue limits (MRL). The Polish law setting such maximum residue limits in foods has been in force since 1993. Maximum residue limits were unified in all EU member states by Commission Regulation (EC) No 839/2008 of 31 July 2008 (Polish Committee for Standardization 2008). The regulation ensures food safety for all consumers and contributes to more intensive international trade. Because of the promotion of healthy lifestyle, detailed monitoring of pesticide residues should include not only fruit and vegetables but also herbs growing in their natural habitat. Pesticide residues monitoring should comprise more and more active substances and foodstuff (Bhanti and Taneja 2005; Wang et al. 2008). In Poland, herb plants are grown on over 20,000 farms, with an area, depending on the species grown, between 0.5 and 2.5 ha and sometimes even between 6 and 10 ha. It means that Poland is one of the leading herb-growing countries in Europe. In Poland, 60 herb species are grown in fields and about 130 species of herb plants grow in their natural habitat.

The aim of this paper is to measure residue concentration of plant protection products in selected herb plants, important in cosmetic, food, and medicine production: *Achillea millefolium* L., *Cichorium intybus* L., *Equisetum arvense* L., *Polygonum persicaria* L., *Plantago lanceolata* L., and *Plantago major* L., all of them growing in their natural habitat close to crop fields.

## Materials and methods

**Fig. 1:**
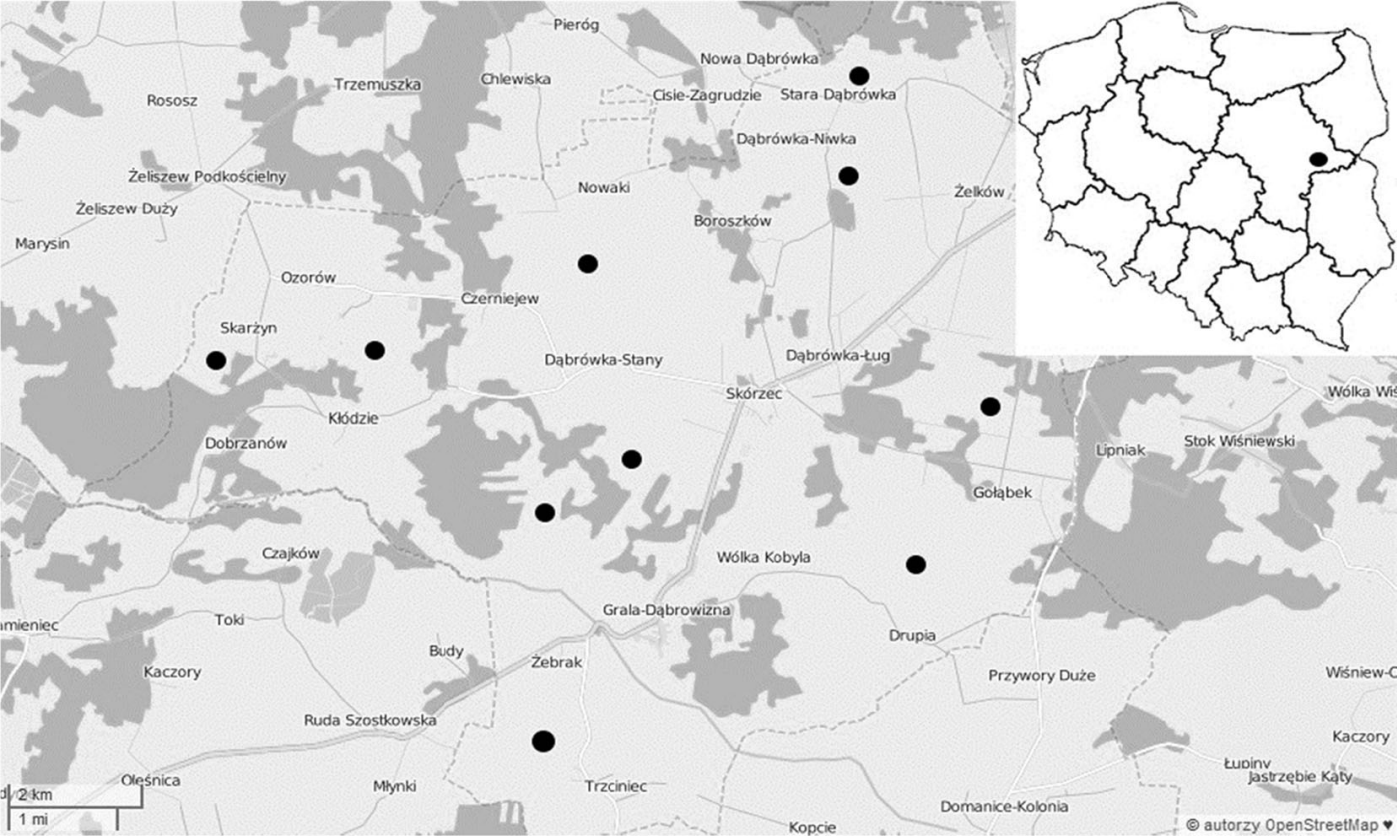
Location of the analyzed samples (openstreetmap.org/#map=12/52.1036/22.1357)

The research material included six species of herbs (*A. millefolium* L., *C. intybus* L., *E. arvense* L., *P. persicaria* L., *P. lanceolata* L., *P. major* L.) sampled 1 and 10 m away from the edges of the winter wheat field and the maize field. The plants came from farms located in the Skórzec commune, the County of Siedlce, in east-central Poland (Fig. 1). Those crops were not grown in an organic farming, but according to recommendations, not only both mineral and organic fertilizers but also plant protection products were used for growing them. The farmers followed the directives on the Council of the European Commission concerning protection of waters against pollution caused by nitrates from agricultural sources, like manure, chemicals, or fertilizers (Council Directive 1991; Regulation of the Minister of Agriculture and Rural Development 2010). Between 1 and 20 July 2014, during the flowering stage, herb plants were harvested, with 30 samples of each species gathered. There were five plants in each sample, which was about 200–300 g DM. The plants, leaves, stalks, and inflorescence were dried at the temperature of 105 °C and ground, pesticide residues being determined with the QuECHERS method. The extraction methods of pesticide residues in herb plants are described by Słowik-Borowiec et al. (2012, 2013). This method was used according to Polish Standard PN-EN 15662:2008 (PN-EN 15662) (Polish Committee for Standardization 2008).

Mass spectrometry (MS) was used to indentify compounds. The mass spectrometer is a universal detector (using ECD, NPD, FPD), with an advantage over other spectrometers which do not differentiate isotopes (Kruve et al. 2008; Lesueur et al. 2008).

Mass spectrometry detection allows identifying compounds with the highest probability, using retention time of analyzed substances and their mass spectra. The QuECHERS method makes it possible to be modified to analyze different compounds from different matrices, using different analytical techniques and equipment (Lehotay et al. 2010). The analysis was carried out by adding to herb samples a mixture of standards at two spiking levels. For each level, the experiment was replicated three times. In the experiment, the SampliQ salts and sorbent packets produced by Agilent Technologies were used. The standards and 10 ml of water were added to herb samples (2 g ± 0.03). For an extraction, acetonitrile solvent (10 ml) was added together with salts: sodium chloride, trisodium citrate, disodium hydrogen citrate, and anhydrous magnesium (IV). Part of the acetonitrile extract was cleaned up using dispersive solid phase extraction (d-SPE) technique, with an addition of PSA salts and sorbents (primary secondary amine), activated carbon, and anhydrous magnesium sulfate. Pesticide residues were determined with the GC-MS/MS method, i.e., gas chromatography mass spectrometry (Shimadzu GCMS-TQ8030EI). MRM database was used for quantization and identification of pesticides. The results (mg kg^−1^) were available on the chromatogram. Percent recovery was calculated as the proportion of the analyte concentration in the fortified sample to the analyte concentration in the standard, using the formula:

$$ F={c}_1/{c}_2\times 100\% $$

(where *c*_1_ is the analyte concentration in the enriched sample, worked out on the basis of peak height measurements; *c*_2_ is the analyte concentration in the standard, and *F* = recovery (Table 1)).

**Table 1:** Recovery of active substances for two spiking levels

Active substances	Standard concentration (mg kg^−1^)	Average concentration (mg kg^−1^)	Mean recovery (%)	Relative standard deviation (RSD) (%)	Standard concentration (mg kg^−1^)	Average concentration (mg kg^−1^)	Mean recovery (%)	Relative standard deviation (RSD) (%)
Diphenylamine	10.5	12.4	118.2	2.7	526	665	112.9	4.3
Tebuconazole	11.8	9.38	79.6	11.3	589	565	107.6	0.8
Chlorpyrifos-ethyl	10.0	12.1	121.2	0.5	500	610	122.1	0.8

The recovery ranged from 79 to 122 %, complying with the EU guidance (SANCO 2012). Altogether, 155 active substances of plant protection products were analyzed in the herb samples together with metabolites and decomposition products (Table 2). The results were compared with maximum pesticide residue limits (MRL) for dried herbs in Poland (The Act on Food Safety and Nutrition 2006).

**Table 2:** Active substances analyses and their detection limits (mg kg^−1^)

Insecticides	Acrinathrin (0.01); aldrin (0.01); alletryna (0.01); azinophos-ethyl (0.01); azinophos-methyl (0.01); 2.4′-DDD (0.01); 2.4′-DDE (0.01); 2.4-DDT + 4.4′-DDD (0.01); 4.4′-DDE (0.01); 4.4′-DDM (0.01); 4.4′-DDMU (0.01); 4.4′-DDT (0.01); bifenthrin (0.01); bromophos-ethyl (0.01); bromophos-methyl (0.01); bromopropylate (0.01); buprofezin (0.01); cyjanofenofos (0.01); chlorfenvinphos (0.01); chlorthiophos (0.01); chlorpyrifos-ethyl (0.01); chlorpyrifos-methyl (0.01); cyfluthrin (0.01); cypermethrin (0.01); deltamethrin (0.01); diazinon (0.01); dicofol (0.01); dichlofenthion (0.01); dichlorvos (0.01); dieldrin (0.01); dimethate (0.01); disulfoton (0.01); endosulfan alfa (0.01); endosulfan beta (0.01); endosulfan SO_2_ (0.01); endrin (0.01); esfenvalerate (0.01); ethion (0.01); ethoprophos (0.01); etrimfos (0.01); fenchlorofos (0.01); fenoxycarb (0.01); fenpropathrin (0.01); fenthion (0.01); fenitrothion (0.01); fenvalerate (0.01); fonofos (0.01); fosalone (0.01); phorate (0.01); formothion (0.01); furathiocarb (0.01); HCB (0.01); α-HCH (0.01); β-HCH (0.01); delta-HCH (0.01); γ-HCH (lindane) (0.01); heptachlor (0.01); endo-heptachlor-epoxide (0.01); heptachlor-exopoxide (0.01); indoxacarb (0.01); isofenphos (0.01); jodophenophos (0.01); kumaphos (0.01); quinalphos (0.01); kwinoksylen (0.01); malathion (0.01); mecarbam (0.01); methacrifos (0.01); methamidofos (0.01); methidathion (0.01); methoxychlor (0.01); mevinphos (0.01); monocrotophos (0.01); omethoate (0.01); paraoxon-ethyl (0.01); paraoxon methyl (0.01); parathion-ethyl (0.01); propargite (0.01); phosalone (0.01); pirimicarb (0.01); pirimiphos-ethyl (0.01); pirimiphos-methyl (0.01); profenofos (0.01); pyridaben (0.01); pyriproxyfen (0.01); sulfotep (0.01); quinalphos (0.01); tebufenpyrad (0.01); terbufos (0.01); tetradifon (0.01); tetrachlorvinfos (0.01); tetrasil (0.01); trichlorfon (0.01); triazophos (0.01)
Fungicides	Azoxystrobin (0.01); benalaxyl (0.01); bitertanol (0.01); boscalid (0.01); bromuconazole (0.01); bupirimate (0.01); chlorothalonil (0.01); chinomethionat (0.01); cyproconazole (0.01); cyprodinil (0.01); diclofluanide (0.01); difenoconazole (0.01); dimethomorph (0.01); dimoxystrobin (0.01); diphenylamine (0.01); epoxiconazole (0.01); fenarimol (0.01); fenbuconazole (0.01); fenhexamid (0.01); fenpropidin (0.01); fenpropimorph (0.01); fludioxonil (0.01); flusilazole (0.01); flutriafol (0.01); folpet (0.01); hexaconazole (0.01); imazalil (0.01); iprodione (0.01); captan (0.01); kresoxim-methyl (0.01); quintozene (0.01); metalaxyl (0.01); myclobutanil (0.01); oxadixyl (0.01); penconazole (0.01); pencycuron (0.01); picoxystrobin (0.01); prochloraz (0.01); procymidone (0.01); propiconazole (0.01); tebuconazole (0.01); tecnazene (0.01); tetraconazole (0.01); tolclofos-methyl (0.01); tolylfluanid (0.01); triadimefon (0.01); triadimenol (0.01); trifloxystrobin (0.01)
Herbicides	Alachlor (0.01); ametryn (0.01); atrazine (0.01); bifenox (0.01); cyanazine (0.01); chlorpropham (0.01); dichlobenil (0.01); dimethachlor (0.01); ethofumesate (0.01); fluchloralin (0.01); fluorodifon (0.01); metribuzin (0.01); metazachlor (0.01); napropamide (0.01); nitrofen (0.01); prometryn (0.01); propachlor (0.01); pendimethalin (0.01); proph (0.01); profluralin (0.01); propyzamide (0.01); terbutryn (0.01); trifluralin (0.01);
Growth retardant	Paclobutrazol (0.01)

## Results and discussion

Three active substances were found in the analyzed herbs: diphenylamine, tebuconazole, and chlorpyrifos-ethyl (Table 3). Those substances are prohibited to be used on herbal plant fields (in compliance with the Minister of Agriculture and Rural Development’s register of authorized plant protection products) (Regulation of the Minister of Agriculture and Rural Development 2010). Pesticide residues were found in herbs growing close to the wheat field, most often 1 m away from it. No residues were found in herbs growing close to the maize field. Out of six plant species, three of them contained pesticide residues, which constituted 50 % of all analyzed samples. In *A. millefolium*, sampled 1 and 10 m away from the wheat field, diphenylamine, a fungicide, was found. The other two herb species *P. lanceolata* L. and *E. arvense* contained residues of two active substances each, diphenylamine and tebuconazole, both being herbicides, as well as tebuconazole and chlorpyrifos-ethyl, which are insecticides. Malinowska et al. (2015) report that tebuconazole was frequently found in cereal grains in 2013. Plants containing more than one residue are exceptionally toxic to humans (Wang et al. 2008). The use of diphenylamine is banned on nearly all agricultural crops (Commission Implementing Regulation EU 2012), while chlorpyrifos-ethyl cannot be used on cereals. The use of plant protection products from outside the EU might be the cause of the presence of those substances in plants. Moreover, some farmers may use those pesticides on the wrong plants or some pesticides might be used even if they are no longer legal in the EU countries now. Residues over maximum limits in crops, and at the same time in plants growing close to them, can depend on many factors, like weather conditions, the way the pesticide was applied, a dose, the number of pests, a kind of disease, and the withdrawal period (Pussemier et al. 2006; Remlein-Starosta et al. 2015).

**Table 3:** Pesticide residues in plant herbs

Plant species	Plant growing			
	Close to a wheat field		Close to a corn maize field	
	1 m	10 m	1 m	10 m
*Achillea millefolium*	Diphenylamine	Diphenylamine	–	–
*Cichorium intybus* L.	–	–	–	–
*Polygonum persicaria*	–	–	–	–
*Plantago lanceolata* L.	Diphenylamine tebuconazole	–	–	–
*Plantago major* L.	–	–	–	–
*Equisetum arvense*	Tebuconazole chlorpyrifos-ethyl	–	–	–

Diphenylamine was found in two analyzed samples of *A. millefolium*, which is 6.6 % of all of them (Table 4). In plants growing 1 m away for the wheat field, the compound was present in much higher concentration (0.084 mg kg^−1^) than in those 10 m away from the field (0.065 mg kg^−1^).

**Table 4:** Plant protection product residues in some herbs (mg kg^−1^)

Plant species	Number of samples	Active substance	Samples with residues		Concentration of residues (mg kg^−1^)		MRL (mg^.^ kg^−1^)
			Number	%	Min.	Max.	
*Achillea millefolium*	30	Diphenylamine	2	6.6	0.01	0.065 (10 m)	0.05
						0.084 (1 m)	
*Equisetum arvense*	30	Tebuconazole	3	10	0.01	0.140 (1 m)	0.05
		Chlorpyrifos-ethyl				0.037 (1 m)	0.1
*Plantago lanceolata L.*	30	Diphenylamine	3	10	0.01	0.060 (1 m)	0.05
		Tebuconazole				0.031 (1 m)	0.05

In both cases, the concentration was higher than maximum residue limits. In 3 out of 30 samples of *E. arvense*, two active substances were found (tebuconazole and chlorpyrifos), which constituted 10 % of the samples. The concentration of those substances was 0.140 and 0.037 mg kg^−1^, respectively. Concentration of tebuconazole, a fungicide, was three times higher than maximum residue limits for herbs (MRL). In the analyzed samples of *P. lanceolata*, 10 % of them contained two active substances, diphenylamine, with a concentration of 0.060, and tebuconazole, with a concentration of 0.031 mg kg^−1^.

In annual reports on plant protection product residues in Poland, there is not much information on residues in herbal plants. The following herb plants, in single samples, are analyzed most often: horseradish, lemon balm, mint, and plantain (2012 and 2013 Report, www.inhort.pl). In India, Rao et al. (2011) did not find pesticide residues in those plants: *Terminalia belerica*, *Terminalia chebula*, *Emblica officinalis*, and *Withania somnifera*, all of them having medicinal properties. In Poland, monitoring of pesticide residues in herbs is very limited probably because those plants are grown by organic farmers. However, it should not be forgotten that most herbal plants grow in natural habitats; therefore, their quality should be always controlled. Medicinal plants monitoring in China and India is much more common than in Poland (Qian et al. 2010; Rao et al. 2011; Zuhang and Gong 2012).

## Conclusions

Out of six species of herbal plants, sampled close to the winter wheat and maize fields, pesticide residues were found in the following three species: *A. millefolium*, *E. arvense*, and *P. lanceolata* L. Plants with those residues mostly grew 1 m away from the winter wheat field.

Two active substances of fungicides and one of insecticides were found: diphenylamine, tebuconazole, and chlorpyrifos-ethyl, respectively, all of them illegal to use in herb growing.

Out of 30 samples of *E. arvense* and *P. lanceolata* L., three of them contained two active substances, which constituted 10 % of all samples of the same species. Two samples of *A. millefolium* contained one active substance, which constituted 6.6 %.

On the basis of the results, it can be said that the bioaccumulation of plant protection product residues differs and depends on the plant species. Out of six analyzed plant species, the following three, not containing residues, could be used as medicinal plants: *C. intybus* L., *P. persicaria*, and *P. major* L. Plants growing in natural habitat, even if far away from crop fields, are not always free from chemical contamination. Because some medicinal plants, no matter where they grow, contain pesticide residues, it is necessary to analyze their content. Pesticide residues monitoring should be intensified in order to eliminate all irregularities in the use of plant protection products.
